# Infection in pulmonary vascular diseases: Would another consortium really be the way to go?

**DOI:** 10.21542/gcsp.2019.1

**Published:** 2019-03-31

**Authors:** Ghazwan Butrous, Alistair Mathie

**Affiliations:** University of Kent, UK

There has been a clear engagement by the medical community with pulmonary hypertension after the approval of targeted therapies and the introduction of more therapeutic modalities in the last 18 years. The increasing number of scientific sessions and conferences was inevitable. Major initial interest was from the developed countries , which concentrated on prevalent etiologies: primary (later called idiopathic) pulmonary arterial Hypertension and secondary to connective tissue disorders – currently both classified as Class I^1,2^. Unfortunately, a lesser consideration was given to other causes of pulmonary hypertension such as secondary to left heart failure (Class II) or hypoxic pulmonary disease (Class III). This is presumably due to both the complexity and the multifactorial etiologies of these causes and the lack of availability of targeted therapies.

**Figure 1.**
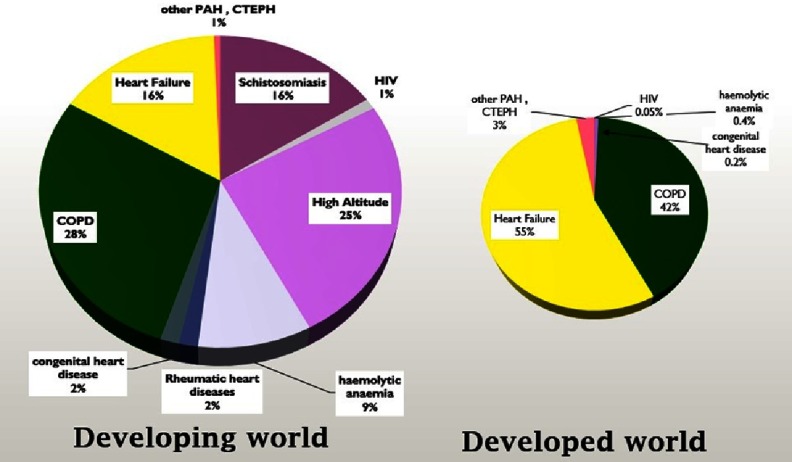
An estimated proportion of the causes of pulmonary hypertension worldwide and the differences in primary causes between developed and developing countries.

On the other hand the developing world, where 6 billion people live, showed less attention to pulmonary hypertension where the pattern and etiologies differ from the developed countries^3,4^. It is difficult to estimate the real extent of pulmonary hypertension in the developing countries due to the lack of proper epidemiological investigations^4^. Figure 1 shows some hypothetical suggestions for the difference of pulmonary hypertension in developing versus developed countries based on the available observations and data. We estimated the risk of subjects developing pulmonary hypertension to be four to six times higher in developing countries compared to developed regions.

A wide variety of infectious diseases can contribute to the causation of pulmonary vascular diseases (PVD) and consequently pulmonary hypertension in the developing world. Over 200 million people (80% of which are in Africa) are affected by schistosomiasis, which may cause PVD in half of them^5^ and the clinical presentation of pulmonary hypertension in 7–15% of them^6,7^. Other Helminthic diseases can induce pulmonary hypertension such as *Wuchereria bancrofti*, a threadlike worm that causes filariasis (elephantiasis)^8,9^. *Clonorchis sinensis* (Chinese liver fluke), which is a widespread parasite in southeast Asia, has been associated with cases of pulmonary hypertension^10^. Some investigators reported other parasitic diseases such as hydatid cysts inducing pulmonary hypertension^11^. Viral infection, mainly HIV which is a global disease, can cause pulmonary hypertension in 0.5 to 5% of infected patients^12^. The condition can be exacerbated by using addictive drugs^13,14^ found in many herbal remedies in the developing regions. Other viral infections, such as human herpesvirus-8, showed evidence of PVD^15,16^. Recent initial reports suggest that fungal infections, like *P. brasiliensis* which causes paracoccidioidomycosis in Brazil, can cause PVD in patients and lab animals^17,18^. Some bacterial infections, such as *B. Pertussis,* may trigger pulmonary hypertension^19^. Other bacterial infections that cause granulomatous reactions in the lungs, like tuberculosis, have been suspected, but not fully evaluated. Indeed, recent cases and communications from Africa and India suggest the potential role of tuberculosis in PVD^20,21^.

There are no real efforts being made to understand the role of infection in PVD despite being one of the major causes of PVD globally, with the exception of some recent work with schistosomiasis and HIV. There are no well-conducted epidemiological studies. Furthermore, co-exposure with more than one infection can be an issue, for example, in some parts of Africa over 50% of patients infected with HIV are co-infected with schistosomiasis^12^.

It is clear that more effort is needed to investigate the role of infection in PVD and consequently pulmonary hypertension for many reasons:

1. Infection is the major cause of pulmonary hypertension globally, making it a prime example of a condition with an unmet medical need.
2. The study of infection will enhance our understanding of the complexity of pulmonary vascular disease pathophysiology.
3. Infection will involve complicated inflammatory and immunological reactions, the two factors that have been increasingly implicated in the pathophysiology of PVD in general^22,23^. Therefore, studying infection in PVD will help to enhance the understanding of the role of inflammation in PVD which helps to assess the role of inflammation in other conditions like the connective tissue diseases that are more complex to investigate.
4. Studying infection in PVD might help the development of new pulmonary hypertension therapies which can also be a valuable clinical tool in the investigation of new potential therapies.

Therefore, more systematic and coherent efforts are needed to study the role of infection in PVD. To help achieve this task, we first need to consider educational efforts to raise awareness globally and to focus the attention of clinicians in identifying and suspecting the diagnosis of pulmonary hypertension secondary to infection. Awareness will support further basic, clinical, and epidemiological research. Second, we need to establish collaborations. The study of infection and PVD needs expertise from different disciplines working together.

**Figure 2.**
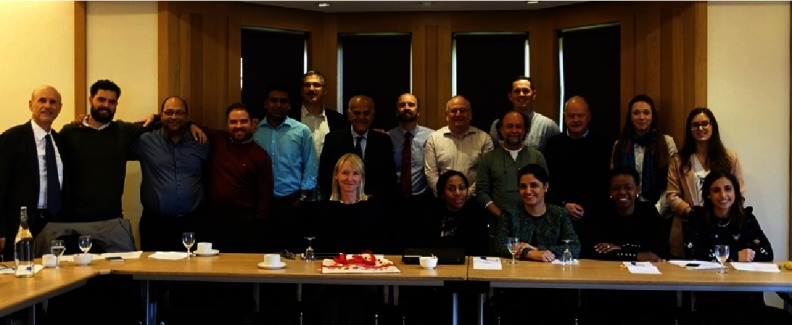
IPVD foundation meeting on 24th October 2018 at Canterbury Cathedral Lodge, Canterbury, United Kingdom.

Thus, we suggest the need of a framework to support this global effort. To move in this direction nineteen experts from basic, clinical, and other disciplines met on the 24th October 2018 at Canterbury Cathedral Lodge in Canterbury (on the grounds of one of the oldest historical buildings in England) to discuss and form a framework to support the global effort of tackling infection and PVD (Figure 2). They proposed the creation of a consortium for infection in Pulmonary Vascular Disease (iPVD Consortium). The aims of this consortium are:

1. Enhance awareness about the role of infection on PVD
  a. By collecting and archiving cases and observations of infection and PVD.
  b. Support special sessions on infection and PVD at international parasitology, immunology, respiratory, cardiology, and specialized pulmonary hypertension conferences.
  c. Write a series of research and review articles on the role of infection and PVD in peer review specialized journals.
  d. Organize regular dedicated conferences on the role of infection and PVD.
  e. Build an iPVD consortium website and social media presence to enhance awareness and communication.
2. Enhance and support collaborative basic and clinical research on the role of infection on PVD.
3. Organize epidemiological and field studies on the role of infection on PVD

The meeting established the basic framework of this consortium based on the above aims and tasks. It will support collaboration on grant applications and exchange ideas and research. The consortium will extend membership to experts from various disciplines to work together.

Thus, the answer to the title of this editorial is “Yes”, we need another consortium to tackle the most forgotten and neglected cause of PVD worldwide. We hope this consortium will help and find support in the years to come to answer many questions about this condition.
